# Odors Attracting the Long-Legged Predator *Medetera signaticornis* Loew to *Ips typographus* L. Infested Norway Spruce Trees

**DOI:** 10.1007/s10886-023-01405-6

**Published:** 2023-01-31

**Authors:** Maria Sousa, Göran Birgersson, Kristina Karlsson Green, Marc Pollet, Paul G. Becher

**Affiliations:** 1 Unit of Chemical Ecology, Department of Plant Protection Biology, Swedish University of Agricultural Sciences, P.O. Box 190, SE 234 22 Lomma, Sweden; 2 Research Institute for Nature and Forest (INBO), Herman Teirlinckgebouw, Havenlaan 88, bus 73, B-1000 Brussels, Belgium

**Keywords:** Biocontrol, Electrophysiology, Host location, Kairomone, Predator–prey interaction, Sustainable forestry

## Abstract

Predatory long-legged flies of the genus *Medetera* are important, but currently understudied, natural enemies of Scolytinae bark beetles such as *Ips typographus*. *Medetera* flies lay eggs on beetle-infested trees, where the developing larvae find their prey, but the chemical cues used by *Medetera* to locate infested trees are currently unknown. To identify odors attracting *Medetera signaticornis,* a species in Europe, headspace samples were collected at several time-points through different stages of *I. typographus* attacks on logs of Norway spruce (*Picea abies*). The headspace samples were analyzed using combined gas chromatography and mass spectrometry (GC–MS), and gas chromatography coupled with electroantennographic detection (GC-EAD) to determine compounds that stimulate *M. signaticornis* antennae. Antennae of *M. signaticornis* males and females were found to detect (–)-*cis*-verbenol, ( +)-*trans*-verbenol and myrtenol, which are known to be produced by bark beetles. Antennal responses were also observed for verbenene, isoterpinolene, α-pinene oxide, camphor, pinocamphone, terpinene-4-ol, myrtenal, borneol, α-terpineol, geranyl acetone, and verbenone, which are primarily produced by microorganisms, and α-pinene, α-fenchene, β-pinene, camphene, 3-carene, limonene, γ-terpinene, and terpinolene, known spruce tree compounds. In field experiments testing two synthetic blends containing 18 antennal active and two additional compounds 2-methyl-3-buten-2-ol and ipsdienol we observed significant attraction of *M. signaticornis* within 24 h. These attractive blends can form the basis for development of *Medetera* monitoring lures for use in future forest and pest management.

**Supplementary Information:**

The online version contains supplementary material available at 10.1007/s10886-023-01405-6.

## Introduction

Norway spruce (*Picea abies* (L.) Karst.), an ecologically and economically important conifer species, is increasingly being threatened by fungal disease (e.g., root and butt rot disease caused by *Heterobasidion* spp.) (Gunulf et al. 2013; Gomez-Gallego et al. 2022) and insect pests (Hannerz et al. 2002; Romashkin et al. 2020; Hlásny et al. 2021). One of the most severe pests on Norway spruce is the Eurasian eight-spined spruce bark beetle (*Ips typographus* Linnaeus) (Coleoptera: Curculionidae, Scolytinae) (Grégoire et al. 2015). Within the coniferous forest ecosystem, *I. typographus* feeds on dead or dying spruce trees and contributes to recycling of nutrients (Edmonds and Eglitis 1989). However, weather conditions such as strong wind, warm temperature and low rainfall can cause mass development of *I. typographus* in windthrown or draught-stressed spruce trees, and in consequence of a high beetle abundance even healthy trees get attacked (Rouault et al. 2006; Kärvemo and Schroeder 2010; Stadelmann et al. 2014). When colonizing living spruce trees, *I. typographus* uses an aggregation pheromone that facilitates mass attack (Birgersson et al. 1984; Schlyter et al. 1987) and introduces a blue-staining symbiotic fungus that metabolizes tree defense compounds toxic to the beetle (Hammerbacher et al. 2013; Wadke et al. 2016; Zhao et al. 2019). In addition, *I. typographus* performs better in warm temperatures, and physiological models predict more frequent outbreaks in European *Picea* forests due to climate change (Marini et al. 2017; Bentz et al. 2019). There is therefore a pressing need to find efficient and sustainable methods to control bark beetles.

Understanding chemo-ecological aspects underlying host finding and infestation of trees by the beetles as well as subsequent trophic interactions might facilitate the development of pest management methods by use of semiochemicals or natural enemies. Tree volatiles serve as signals in host recognition of bark beetles (Seybold et al. 2006). In coniferous trees, terpenoid compounds (primarily volatile mono- and sesquiterpenes or less-volatile diterpenes) are characteristic constitutive or inducible defense chemicals, respectively (Keeling and Bohlmann 2006; Martin et al. 2003). The emission of these compounds can increase as response to biotic and abiotic stress such as the attack by wood boring insects, high temperature or mechanical damage (Ghimire et al. 2016; Juráň et al. 2017; Holopainen et al. 2018).

Several natural enemies of bark beetle, such as parasitoids and predators, are attracted to bark beetle-infested trees by volatile chemical cues that originate from the host tree (e.g., mono- and sesquiterpenes), from bark beetles themselves (e.g., *I. typographus* produced oxygenated hemi- and monoterpene alcohols), and/or from associated microorganisms which the beetles transfer to the host trees and their offspring developing inside the galleries (e.g., oxygenated monoterpenes) (Leufvén et al. 1984; Leufvén and Birgersson 1987; Pettersson 2000; Kandasamy et al. 2016, 2021). Natural enemies can significantly reduce bark beetle populations and are considered to be environmentally safe and sustainable control agents (Wermelinger 2002; Kenis et al. 2007; Wegensteiner et al. 2015).

An important group of predator species of bark beetles are flies of the genus *Medetera* (Diptera: Dolichopodidae). At present these are not actively applied or considered as biocontrol agents in forest management. *Medetera* adults feed on a wide array of small invertebrates (Ulrich 2004), but the larvae of most tree trunk-dwelling *Medetera* species depend on bark beetles for their development. The adult long-legged flies can be observed on tree trunks from early spring throughout the whole summer and up to the first frost. *Medetera* females have been observed to oviposit in newly infested trunks shortly after infestation by bark beetles (Nicolai 1995; Wermelinger 2004). On infested trees, females inspect the bark surface with their ovipositor and lay their eggs near the entrance of bark beetle galleries. A few days later, the newly emerged larvae migrate into the galleries and start feeding on beetle eggs and larvae, and on pupae or newly emerged, callow bark beetle adults that are still concealed in the galleries and pupal chambers (Beaver 1966; Bickel 1985). To locate bark beetle-infested trees, the adult flies use volatile chemical cues (Hulcr et al. 2005, 2006). Information on the specific compounds required for host detection is scarce, but it is known that some *Medetera* spp., such as *M. setiventris* Thuneberg and *M. melancholica* Lundbeck, are attracted to the *I. typographus* aggregation pheromone, which consists of a mixture of (*-*)-*cis*-verbenol and 2-methyl-3-buten-2-ol (Hulcr et al. 2005, 2006). According to Hulcr et al. (2006), the number of *M. setiventris* attracted to traps increases when *I. typographus* aggregation pheromone is combined with ipsdienol. Furthermore, *M. signaticornis* Loew has been shown to be attracted to a mixture of host tree compounds such as α-pinene, β-pinene, camphene, and limonene dissolved in ethanol (Rudinsky et al. 1971), while α-pinene has been shown to stimulate oviposition in females of *M. aldrichii* Wheeler and seems to guide newly emerged larvae to prey gallery entrances (Fitzgerald and Nagel 1972).

*Medetera signaticornis* is described as one of the most common *I. typographus* predators in Europe (Ounap 2001; Wermelinger 2002), which makes it a good candidate species for use in a future biocontrol strategy. In this study, we tested the hypothesis that *M. signaticornis* adult flies use multiple semiochemicals to detect bark beetle-infested Norway spruce trees. In order to identify key compounds that attract *M. signaticornis* to infested spruce, we: *i)* compared the volatilome of bark beetle-infested standing trees, bark beetle-infested cut trees and non-infested spruce trees over time; *ii)* identified odor compounds from infested trees eliciting electroantennographic responses on the antennae of *M. signaticornis* adults; and *iii*) tested the effectiveness of synthetic olfactory-active compound blends under field conditions.

## Material and Methods

### Insects

Males and females of *M. signaticornis* were collected with a mouth aspirator from bark beetle-infested spruce trees (*Picea abies*) at two different sites (1 and 2, 57.150°N, 14.765°E and 57.127°N, 14.780°E, respectively) close to the SLU field research station in Asa, Småland province, Sweden, between May and August during 2018 and 2019. The average of the daily maximum temperature between May and August 2018 and 2019 at Asa was 24.3 ± 4.5 °C and 20.6 ± 3.5 °C, respectively, while the average daily precipitation during the same period was 1.65 ± 4.6 mm and 3.55 ± 8.1 mm (more detailed weather data can be accessed from the Asa weather station, Anon (2023)). Collected flies were placed individually in glass vials with humidified filter paper and transported to the laboratory, where they were kept starved at 4–8 °C until electrophysiology studies, which were carried out within a week collection of the flies.

### Volatile Collection

Odor samples for chemical and electrophysiology analysis were collected from non-infested standing trees, infested standing trees, and infested cut trees at the two sites during 2018. A total of 11 healthy mature standing spruce trees, approximately 40–60 years age, were randomly selected at the two sites. Four of these trees were cut with a chainsaw (three at site 1 and one at site 2), and the fallen trunks and two standing trees at each site were baited with synthetic *I. typographus* pheromones (see below) to induce controlled *I. typographus* attacks. The remaining three trees (one from site 1 and two from site 2) were left without pheromone bait and used as controls.

The bait used to attract bark beetles to both cut and standing trees consisted of single dispensers containing the synthetic *I. typographus* aggregation pheromone (Pheroprax®, BASF, Limburgerhof, Germany). The dispensers were suspended at 3 m height (measured from the bottom of the tree) on the bark of the standing trees, and cut trees. Once *I. typographus* beetles had started excavating galleries in the baited trees, the dispensers containing synthetic bark beetle pheromones were removed and headspace collection was started.

**Fig. 1:**
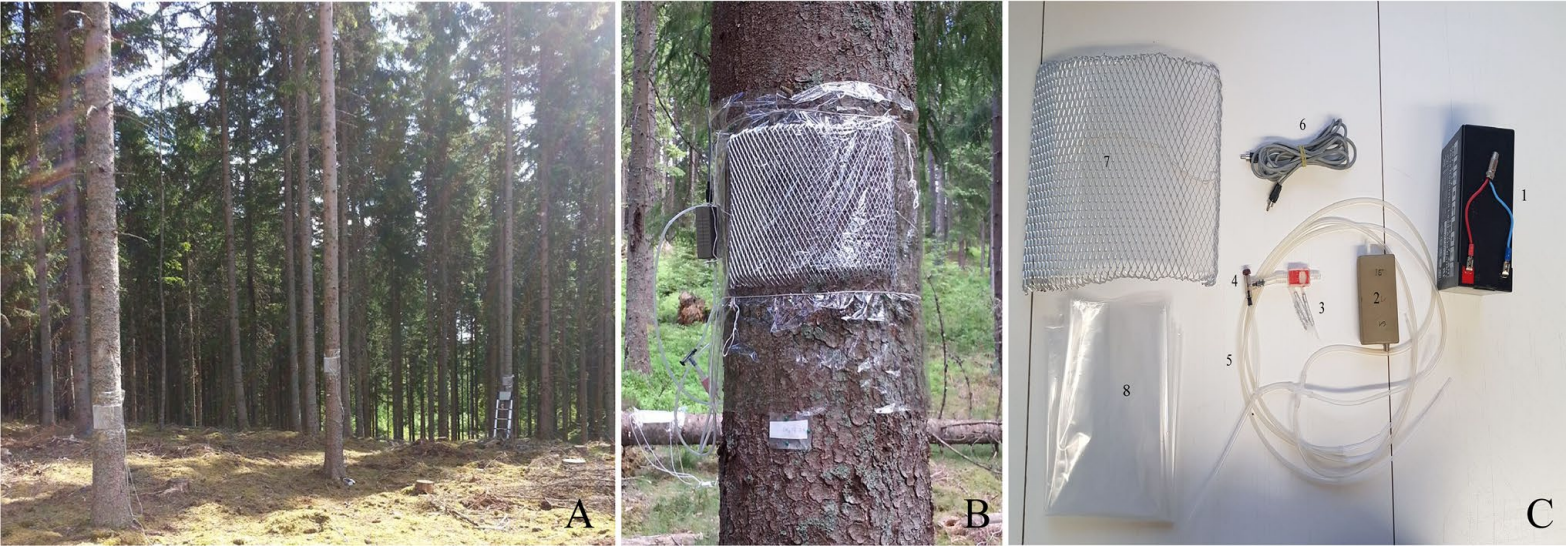
Setup used for headspace collections from logs of Norway spruce trees (*Picea abies*) through the different stages of *Ips typographus* attack. **A**) three sampled standing trees in one of the field sites; **B**) metal grid covered with a polyester roasting bag that was wrapped around the tree bark forming an enclosure for odor collections; **C**) material used: 1. battery; 2. sucking air pump; 3. adsorbent columns (3 × 55 mm); 4. air splitter; 5. Silicone tubing; 6. Cables to connect the battery to the air pump; 7. aluminum grid (area 32 × 32 cm); 8. polyester roasting bag

A curved aluminum grid (area 32 cm × 32 cm, with 1.5 cm distance between grid and bark) was attached to each tree at specific collection points between 1.5 and 2 m tree height (measured from the bottom of the tree), to provide an open space for volatile release (Fig. 1A-C). For the eight infested cut or standing trees, the aluminum grid was affixed to cover the entrance hole(s) of one or two bark beetle galleries. To collect the volatiles emanating from the bark surface, the aluminum grid was covered with a polyester roasting bag (Toppits®, Cofresco Frischhalteprodukte GmbH, Minden, Germany) wrapped around the tree bark, giving an open bark surface area for volatile release of ~ 9 dm^2^. Nylon wire was used to tie the upper and lower edges of the polyester bag to the tree (Fig. 1B). The released volatiles were collected through an adsorbent column (3 × 55 mm PTFE Teflon® tube, inner diameter 3.0 mm, outer diameter 4.0 mm, filled with ~ 30 mg of Porapak Q (mesh 50/80, Waters, Milford, MA, USA)) that had been placed in the open space under the grid before wrapping with the polyester bag. The absorbent column was connected by silicone tubing to a battery-driven membrane pump (KNF NMP830KNDC, KNF, Sursee, Switzerland) and the air drawn through each column was adjusted to a flow rate of 150 mL^.^min^−1^ for 3 h. An additional Porapak Q column was connected to the pump for sampling potential contamination from the air outside the enclosed aluminum grid. Compared to the samples from the bark inside the grid, amounts of compounds trapped outside the grid were neglectable (data not shown). After headspace collection, the polyester bag was cut open and the column was transferred to a clean glass vial. All vials were sealed and transported to the laboratory in a container with ice. In the laboratory, each column was eluted with 500 µL of pentane (*puriss p.a*, Sigma-Aldrich, Saint Louis, MO, USA) and the eluate was stored at -20 °C. Headspace collections from the same collection points on the same experimental trees (C1-C7) were made approximately every 10–15 days over a period of two months (from 10^th^ May for site 1 or 25^th^ May for site 2 up to 25^th^ July), until the new generation of bark beetles began to emerge. The starting time of headspace collections between sites differed slightly because bark beetles were active earlier at site 1 compared to site 2. However, collections C1 to C6 from sites 1 and 2 correspond to similar stages of bark beetle attack. Details about collection dates and climatic conditions can be found in the Supplementary Table 1. In total, we were able to collect and analyze 12 samples from non-infested Norway spruce trees, 26 samples from infested standing trees, and 25 samples from infested cut trees.

### Analysis by Gas Chromatography Coupled to Mass Spectrometry (GC–MS)

All odor samples were concentrated to 100–150 µL and combined with 10 µL of heptyl acetate (100 ng µL^−1^) as internal standard. Then 2 µL of each sample were injected by auto-injector (G4567A) into a gas chromatograph (7890B, GC) with mass spectrometry detection (5977A MS) (all Agilent Technologies, Santa Clara, CA, USA). The GC was equipped with a 60 m × 0.25 mm fused silica column coated with DB-Wax (polyethylene glycol, df = 0.25 µm, Agilent Technologies). Helium was used as the mobile phase, with a constant flow rate of 35 cm^.^s^−1^. The temperature program increased from 40 °C (3 min hold) at 8 °C^.^min^−1^ to 225 °C, which was held for 10 min. Electron impact mass spectra were obtained at 70 eV. All compounds were tentatively identified by comparison of the mass spectra obtained against: *i*) reference mass spectra from our custom library (based on spectra of standards analyzed on our GC–MS devices), supplemented with commercially available MS libraries (NIST, Wiley), and *ii*) Kováts retention index (RI) with reference to public RI libraries (PheroBase) (El-Sayed 2016).

### Insect Preparation and Analysis by Gas Chromatography Coupled to Electroantennographic Detection (GC-EAD)

To identify odor compounds from infested trees eliciting electroantennographic responses on antennae of *M. signaticornis* adults, the flies were gently inserted into a disposable plastic pipette tip with the narrow opening cut wider to let the fly’s head pass through while retaining the thorax and abdomen inside the tip. A piece of glass wool was stuffed into the tip behind the insect body to immobilize the fly. Two glass electrodes were filled with Beadle-Ephrussi Ringer solution and one was inserted into one of the fly’s eyes (indifferent electrode), while the other electrode was connected to a fly’s antenna and mounted on a 10 × preamplifier probe (Ockenfels Syntech GmbH, Buchenbach, Germany) attached to an Intelligent Data Acquisition Controller (IDAC-2, Ockenfels Syntech).

For each GC-EAD analysis, 2 µL of odor sample with internal standard were used (five replicates per fly sex). The GC column and temperature program applied were similar to those used for GC–MS analysis. Hydrogen was used as a mobile phase, at 45 cm^.^s^−1^. At the GC effluent, 4 psi of nitrogen was added and split 1:1 in a Gerstel 3D/2 low dead volume four**-**way cross (Gerstel GmbH & Co KG, Mülheim, Germany) for simultaneous flame ionization detection and EAD recording of the separated compounds. The compounds eluting from the effluent capillary for EAD were mixed with charcoal-filtered humidified air (1.5 L^.^min^−1^) in a glass tube (length 10 cm, inner diameter 6.7 mm) and released close to the prepared fly antenna. A compound was categorized as biologically active if it elicited a reproducible response in the fly antenna. All flies used for the electrophysiological studies were transferred from the plastic pipette tips to vials with 76% ethanol for subsequent confirmation of species identity by morphological analysis.

### Field Trapping Experiments

To investigate whether *M. signaticornis* adults can be effectively attracted and collected using volatiles released by infested trees, we performed a study with available synthetic chemicals comprising 18 compounds categorized as active in GC-EAD and two additional compounds, 2-methyl-3-buten-2-ol and ipsdienol, reported to be involved in attraction of *Medetera* spp. (Hulcr et al. 2005, 2006).

For potential pest management application in the future it might be beneficial to attract *Medetera* flies to infested trees at the early stage of the beetle attack. We therefore selected synthetic mixtures of volatiles related to an early infested spruce tree for our trapping experiments. Two different quantitative compositions of the synthetic chemicals were tested: *i)* a 1:1 mix in which GC-EAD active and additional compounds were prepared in equal proportions, and *ii)* a natural mimic in which GC-EAD active and additional compounds were prepared according to the amounts released from 1 000 dm^2^ of an early infested standing Norway spruce tree (Table 1). Hexane (≥ 97%, Merck, Germany) was used as diluting solvent and as control.

**Table 1:** Purity and amounts of synthetic compounds used in the field traps

Compounds	CAS number	Purity %	Amount of compound in bait (µg/ 2 mL; w/v)	
			Mix 1:1	Natural mimics
2-methyl-3-buten-2-ol	115–18-4	≥ 97%	100	8
( ±)-α-pinene	80–56-8	≥ 98%	100	3 432
(1*S*)-(–)-β-pinene	18,172–67-3	≥ 99%	100	3 462
camphene	79–92-5	≥ 95%	100	181
terpinolene	586–62-9	≥ 90%	100	129
( ±)-camphor	76–22-2	≥ 95%	100	8
(–)-terpinen-4-ol	20126–76-5	≥ 95%	100	12
(1*R*)-(–)-myrtenal	57526–63-3	≥ 98%	100	3
(–)-*cis*-verbenol	18881–04-4	≥ 95%	100	12
( +)-*trans*-verbenol	473–67-6	50%	100	16
(–)-borneol	507–70-0	≥ 97%	100	5
(1*S*)-(–)-verbenone	1196–01-6	≥ 99%	100	4
(1*R*)-(–)-myrtenol	19894–97-4	≥ 97%	100	6
geranyl acetone	3796–70-1	≥ 98%	100	4
α-terpinene	99–86-5	≥ 94%	100	13
γ-terpinene	99–85-4	≥ 98.5%	100	10
(*R*)-( +)-limonene	5989–27-5	≥ 98%	100	252
(*S*)-(–)-limonene	5989–54-8	≥ 92%	100	252
α-terpineol	10482–56-1	≥ 98%	100	34
ipsdienol	35628–00-3	≥ 90%	100	2

Trapping experiments were carried out between June and August 2019 at two different locations in Sweden affected by continuous spruce bark beetle outbreaks. These were Perstorp in Halland County (56.494°N, 13.210°E) and Asa (57.150°N, 14.765°E). At both locations, we used sticky traps that consisted of cardboard rectangles (90 cm × 30 cm) with a printed spruce bark pattern covered with transparent sticky plastic foils and fixed vertically 30–40 cm above the ground on a wooden stick (Supplementary Fig. 1). The distance between traps was around 10 m. We used two traps per synthetic mixture, with three replicate sets during the experimental period. The traps were baited with dispensers consisting of a roll of dental cotton (0.5 cm outer diameter, 3.5 cm length; 2.75 cm^3^; DAB Dental, Upplands Väsby, Sweden) that was impregnated with 2 mL of a synthetic blend and sealed inside a low-density polyethylene sachet (LDPE; 60 × 60 mm; thickness 50 µm) (Rajapack, Gothenburg, Sweden). The dispensers were fixed in the center of the sticky traps. After 24 h, the traps were checked and the numbers of *Medetera* flies and *I. typographus* beetles were counted. *Medetera* flies were separated by sex based on the hypopygium (male genital apparatus) (Supplementary Fig. 2). The *Medetera* flies were transferred to vials with 76% ethanol for subsequent identification.

### Statistical Analysis

All statistical analyses were performed in R studio (version 1.3.959) (Team 2020). Square roots of mean amounts of compounds were plotted in a heatmap using the function *ggplot* from the package *ggplot2*. To test for significant qualitative and quantitative differences between: *i)* volatile profiles collected from the different treatments (non-infested, infested cut, and infested standing trees); and *ii*) overall volatile profiles from infested samples over time, we used Permutational Multivariate Analysis of Variance (*PERMANOVA,* based on Bray-Curtis distances calculated from amount of compounds, 999 permutations) and pairwise *PERMANOVA* with *Bonferroni* correction for multiple testing, using the functions *adonis* and *pairwise.factorfit* from the *vegan* package in R (Oksanen et al. 2014). To visualize the differences in overall data collected, we used two different ordination methods: non-metric multidimensional scaling (*NMDS*, based on Bray–Curtis distances) from the package *vegan* with the function *metaMDS* (Bühler 2012) and principal component analysis (*PCA*) from the package *ade4* with the function *fviz_pca_ind* (Oksanen et al. 2015). To identify groups of compounds found more often in one treatment or at one site compared with another, we applied multi-level pattern analysis with the *multipatt* function from the *indicspecies* package (De Cáceres et al. 2010).

We used one-way analysis of variance (*ANOVA*) with the function *aov* to compare the number of flies collected in the traps with synthetic blends. We performed post-hoc tests with *emmeans* for pairwise multiple comparisons.

## Results

### Volatile Collection and Analysis

**Fig. 2:**
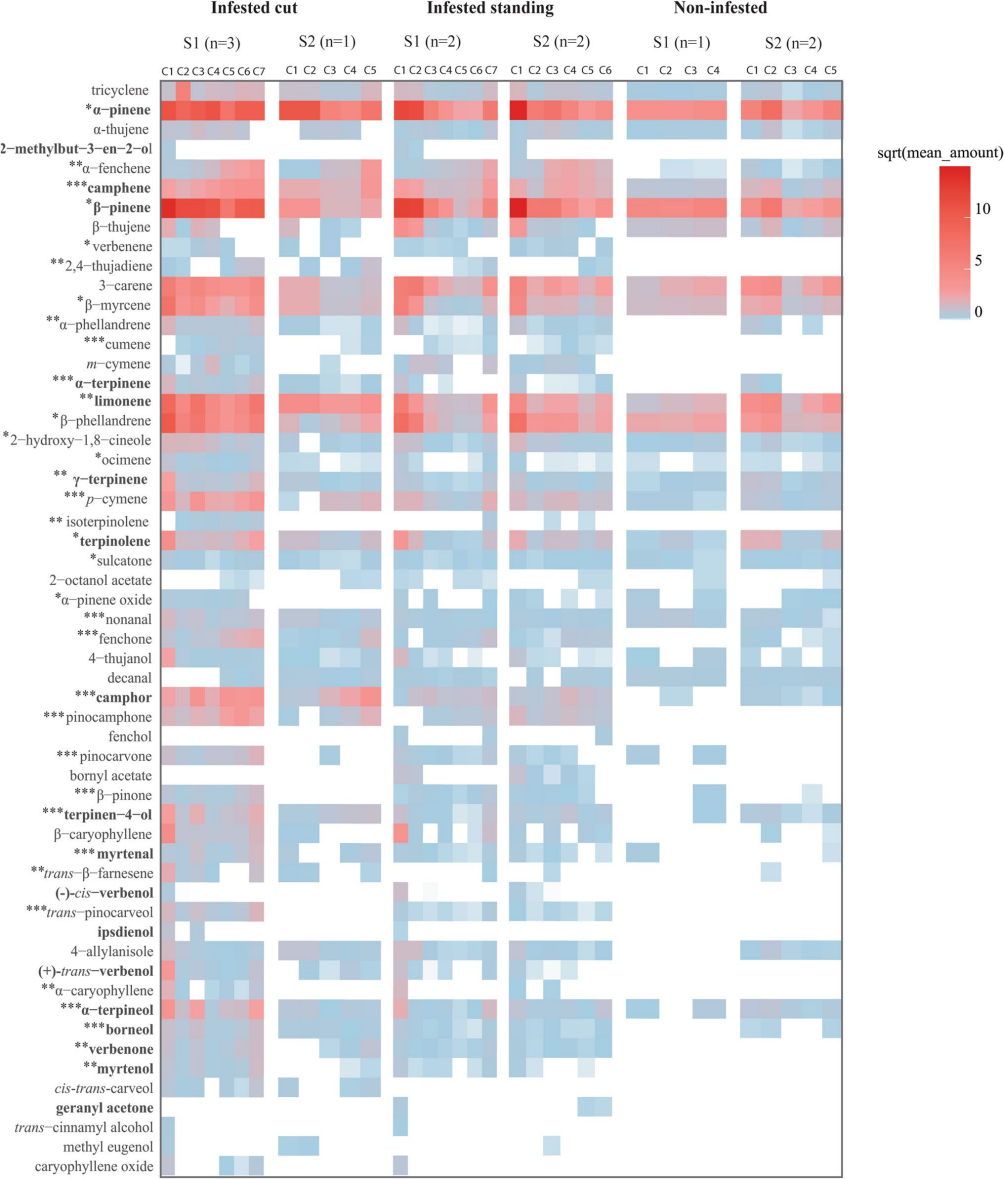
Abundance (square root of the mean amount released per surface area and time (ng(dm^2^^.^s)^−1^)) of compounds detected in the headspace samples collected from cut Norway spruce trees (*Piceae abies*) infested by spruce bark beetles (*Ips typographus*), standing infested or non-infested trees. Odors were sampled from trees at two forest sites (S1 and S2) in up to seven sequential collections (C1 to C7) from 10^th^ May (for S1) or 25^th^ May (for S2), respectively, to 25^th^ July 2018. The dates for the first headspace collections C1 differed slightly between sites as bark beetles were earlier active at S1 compared to S2. However, collections C1 to C6 from sites 1 and 2 correspond to similar stages of bark beetle attack. The compounds are listed in the order of their GC retention time. Asterisks (*) indicates significant differences in the abundance of compounds between the treatments (*Multipatt*, *P ≤ 0.05; **P < 0.01; ***P < 0.001); n describes the number of trees used in each site per treatment. Compounds in bold have been effectively identified with synthetic standards

**Fig. 3:**
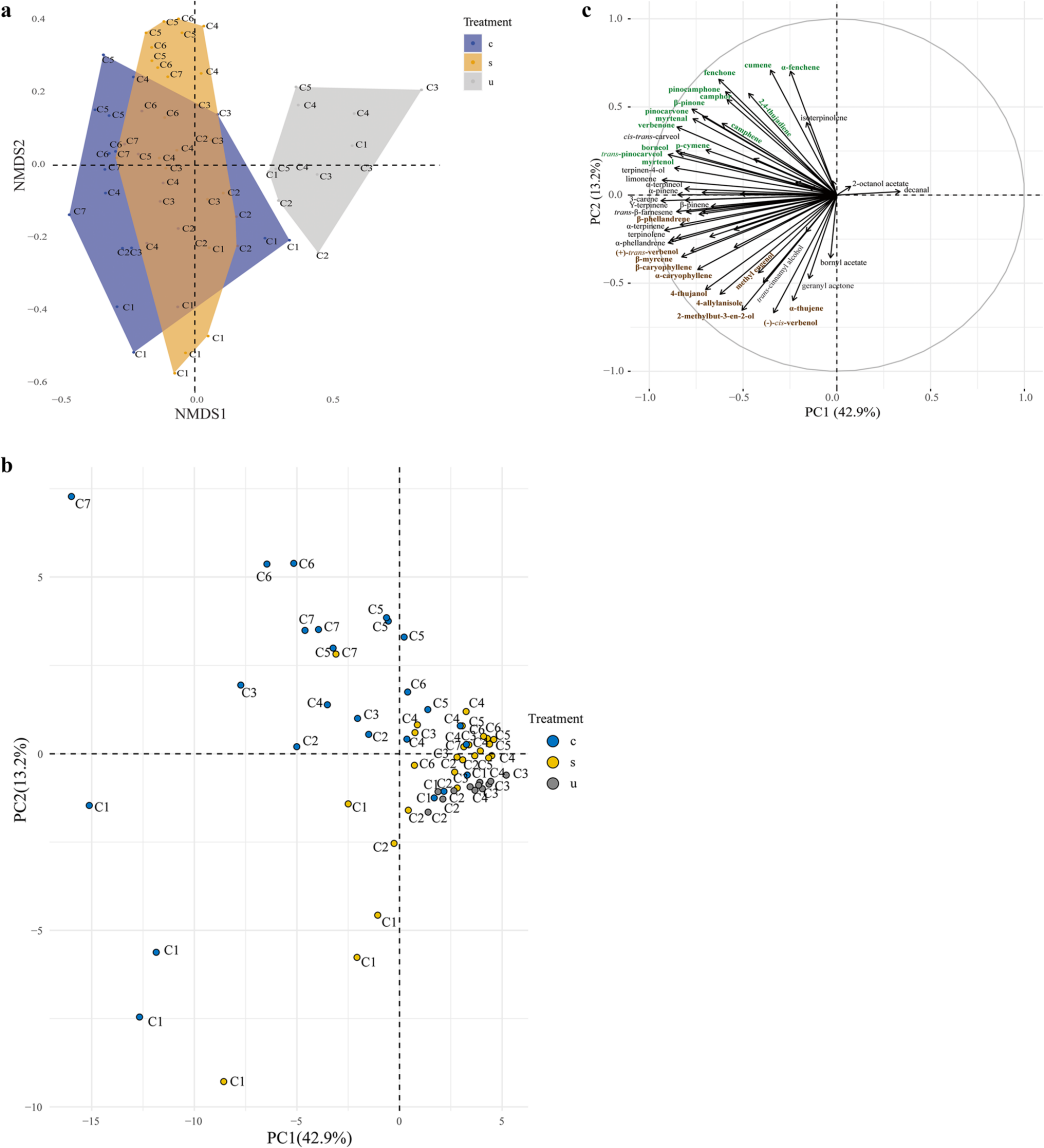
Comparison between the overall volatile profiles found in the headspace samples collected from cut Norway spruce trees (*Piceae abies*) infested by bark beetles (*Ips typographus*) (c), standing infested (s) or non-infested trees (u) over time. **A**) Non-metric multidimensional scaling (*NMDS*) with two synthetic axes and a stress value lower than 0.09.** B** and **C**) Principal component analysis (*PCA*) with *PC1* and *PC2* summarizing 56.1% of the variance of the dataset. Individual samples from the differently treated trees illustrated in **A** and **B** are labeled with different colors representing bark beetle-infested cut trees (blue, *n* = 25), bark beetle infested standing trees (yellow, *n* = 26) and non-infested trees (grey, *n* = 12). Numbers on the color-labeled points C1-7 designate the order of individual collections within the three treatments. Samples that are positioned close to each other have similar volatile profiles. The variable plot **C**) represents the contribution of individual compounds to the main variance in the dataset. Positively correlated compounds are grouped in the same quadrant; negatively correlated compounds are grouped in opposite quadrants. Compounds with long distance from the origin (long arrows) strongly contribute to the samples loaded in the same quadrant. Compounds represented in brown contribute significantly to the overall odor profiles at the beginning of the bark beetle attack (C1-C2) and compounds represented in green contribute significantly to the overall odor profiles at the end of bark beetle attack (C5-C7) (*Multipatt*, *P ≤ 0.05; **P < 0.01; ***P < 0.001)

A total of 118 compounds were found in 63 headspace collections from non-infested trees, infested standing trees, and infested cut trees (Supplementary Table 2). Figure 2 shows 56 of these compounds, 37 that were tentatively identified and 19 that were effectively identified. The overall volatile profiles differed significantly between non-infested and infested samples (*PERMANOVA*, P = 0.001). Within the samples from infested trees, the overall volatile profiles also differed between standing and cut trees (pairwise *PERMANOVAs,* all *Bonferroni*-corrected, P = 0.01) and changed significantly over time (*PERMANOVA*, P = 0.001). In contrast, volatile profiles of samples from non-infested trees did not differ over time (*PERMANOVA*, P > 0.1). When represented with *NMDS*, the overall headspace samples from non-infested trees were clearly separated from those from infested trees, while the samples from infested standing and infested cut trees were grouped together (Fig. 3A). This indicates that headspace samples collected from infested standing and cut trees were more similar, both qualitatively and quantitatively, than headspace samples collected from non-infested trees. Similar results were obtained in the *PCA* plot (Fig. 3B). However, principal component 1 (*PC1*), which discriminated between the treatments (42.9%), also showed that headspace samples grouped at the left-hand side of* PC1* (mainly samples of from infested cut trees) displayed much higher amounts of compounds than samples grouped at the right-hand side of* PC1* (Figs. 2 and 3B).

The overall volatile profile for non-infested trees, compared with infested trees differed in terms of amounts of released compounds (e.g., camphene, cumene, α-terpinene, p-cymene, α-terpineol, terpinen-4-ol, camphor, pinocamphone, borneol, and verbenone) (Fig. 2).

Among the headspace samples from infested trees, samples from cut trees contained significantly more hydrocarbon monoterpenes (e.g., camphene, isoterpinolene, and α-terpinene), oxygenated monoterpenes (e.g., camphor, borneol, fenchone, pinocamphone, terpinen-4-ol, pinocarvone, myrtenal, and *trans*-pinocarveol), and aldehydes (e.g., nonanal) compared with samples from standing trees (*Multipatt*, *P* < 0.05) (Fig. 2, Supplementary Table 3). Volatile compounds such as fenchol, bornyl acetate, and geranyl acetone were only found in samples collected from standing trees, while caryophyllene oxide and *cis*–*trans* carveol were only found in samples collected from cut trees (Fig. 2).

Pairwise comparisons of infested samples collected at different time-points showed that samples collected during late bark beetle attack phases (C5-C7) differed significantly from samples collected during earlier attack phases (C1-C2) (pairwise PERMANOVA, all Bonferroni-corrected, P = 0.01). This was confirmed by the *NMDS* and *PCA* plots (Fig. 3A, B), with samples collected at the beginning of the attack (C1-C2) mainly situated in the lower, negative part of both *NMDS*2 (Fig. 3A) and *PC2* (Fig. 3B). On the other hand, samples collected later during the attack (C5-C7) clustered more in the upper positive part of both *NMDS*2 (Fig. 3A) and *PC2* (Fig. 3B).

The variable correlation plot resulting from the *PCA* (Fig. 3C) illustrates the contribution of each compound to the variation in overall dataset (including all samples collected from non-infested and infested trees at different time-points). Compounds in the lower part of *PC2* (brown in Fig. 3C), such as α and β-caryophyllene, α-thujene, β-myrcene, and β-phellandrene, were emitted in larger amounts at the beginning of bark beetle attack, while compounds in the upper part of *PC2* (green in Fig. 3C), such as pinocarvone, myrtenal, fenchone, β-pinone, and verbenone, were emitted in larger amounts at the end of the attack (*Multipatt*, both *P* < 0.05). This indicates that emission of hydrocarbon monoterpenes was high at the beginning of bark beetle attack and that emission of oxygenated monoterpenes was high at the end of the attack (Fig. 2; Supplementary Table 3). The shift in the overall volatile composition of infested trees seemed to occur during the first 30–40 days after bark beetle attack was initiated.

We also found a significant interaction between treatment and site (*PERMANOVA*, P = 0.02). The reason was that odor samples from infested cut trees collected at Site 1 contained higher amounts of hydrocarbon and oxygenated monoterpenes than samples from infested cut trees at Site 2 (*Multipatt*, *P* < 0.05) (Fig. 2, Supplementary Table 3).

### GC-EAD Responses by *Medetera signaticornis* to Early-infested Standing Spruce Odor Samples

**Fig. 4:**
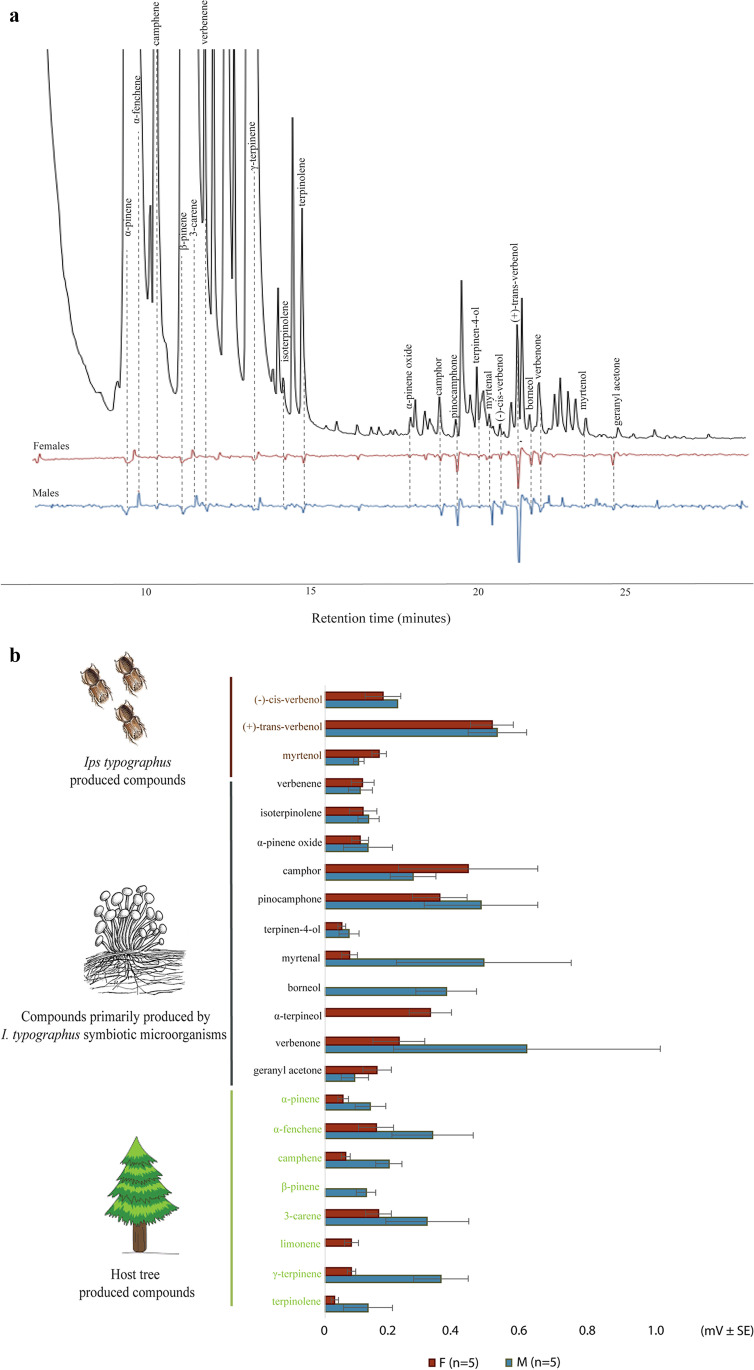
GC-EAD responses of *M. signaticornis* fly antennae stimulated with odors collected from early infested Norway spruce tree. **A**) Chromatogram and electroantennograms showing the antennal stimulation of a female and a male fly antenna in response to compounds eluting from the GC. Dotted lines connect antennal responses with chromatogram peaks of active compounds. **B**) Mean response (mV ± SE) to the active compounds organized according to their source. Compounds represented in brown font are produced by the bark beetle *Ips typographus*, compounds in black font are produced by the *I. typographus* associated microorganisms and compounds in green font are produced by the Norway spruce tree (*Picea abies*). (F) females and (M) males

GC-EAD analysis revealed that 22 active compounds from early-infested spruce trees elicited similar antennal responses in both *M. signaticornis* males and females. The active compounds were identified and divided into three groups according to their primary source of origin (Fig. 4A, B). The first group comprised (–)-*cis*-verbenol, ( +)-*trans*-verbenol, and myrtenol, which are known as compounds produced by *I. typographus* (Birgersson et al. 1984; Birgersson 1989). The second group comprised isoterpinolene, α-pinene oxide, camphor, pinocamphone, terpinen-4-ol, myrtenal, borneol, α-terpineol, verbenone and geranyl acetone, all of which except verbenene are oxygenated monoterpenes that are known to be primarily produced by microorganisms associated with *I. typographus* (Leufvén et al. 1984, 1988; Kandasamy et al. 2021). The third group comprised α-pinene, α-fenchene, β-pinene, camphene, 3-carene, limonene, γ-terpinene and terpinolene, which are hydrocarbon monoterpenes produced by the spruce host tree (Phillips and Croteau 1999; Keeling and Bohlmann 2006). We found that α-terpinene elicited antennal responses in some female *M. signaticornis*, but not consistently in all replicates*.* However, the antennal activity of this compound was confirmed later with a synthetic standard (data not shown).

### Field Trapping Experiments

Traps with either of the two synthetic blends of chemicals (mix 1:1 and natural mimic) caught significantly more *Medetera* flies than the hexane control (F = 4.3; df = 2, 18; *P* < 0.05) (Table 2). The number of flies trapped was similar for the two synthetic blends. Approximately 71% of the flies identified from traps with synthetic blends were *M. signaticornis* females, 27% were *M. signaticornis* males, and 2% were *M. ambigua* Zetterstedt (Table 2).

**Table 2:** Total number of *Medetera* species and *I. typographus* collected in the field traps during a 24 h trapping experiment

	Control	Mix 1:1	Natural mimics	P value
Total number of *Medetera*	54^b^	158^a^	132^ab^	F = 4.3; df = 2,18; *P* < 0.05
*M. signaticornis*	23F; 7 M	80F; 32 M	73F; 26 M	
*M. ambigua*		2F; 1 M	1F	
Unidentified	24	43	32	
Total number of Scolytidae				
*I. typographus*	2^b^	77^a^	106^a^	F = 9.8; df = 2,18; *P* < 0.005

Different small letters (a,b) within a row indicate significant differences between the total number of *Medetera* species or *I. typographus* individuals found in traps with the three different baits according to post-hoc tests following one-way *ANOVA*. F-values provide a measure for variation between samples, df abbreviates the degrees of freedom and *P* < 0.05 provides a measure for significant difference between means. Note that not all specimens could be identified to species level due to body damage caused from the sticky traps. (F) Females or (M) males refers to the total number of specimens collected from each sex

The number of *I. typographus* beetles was also significantly higher for both synthetic blends compared with the control (F = 9.8; df = 2,18, *P* < 0.005). The traps baited with natural mimic collected slightly more bark beetles than the traps with the 1:1 mix, but the difference was not statistically significant.

## Discussion

Our aim in this study was to identify key compounds that attract the bark beetle predator *M. signaticornis* to bark beetle-infested Norway spruce trees. In field trials, we demonstrated that *M. signaticornis* females and males were attracted to synthetic blends of compounds associated with bark beetles, their symbiotic microorganisms, and host trees. Analyses of headspace samples from infested Norway spruce trees showed that headspace composition and compound concentration varied depending on the time-point of collection, apparently following different stages of bark beetle attack (early, late).

More specifically, our analyses revealed that headspace samples from early-infested trees contained high amounts of hydrocarbon monoterpenes such as α and β-caryophyllene, α-thujene, β-myrcene, and β-phellandrene, while headspace samples from late-infested trees were mainly dominated by oxygenated monoterpenes such as pinocarvone, myrtenal, fenchone, β-pinone, and verbenone. Previous studies have also found that headspace samples from bark beetle-infested logs contain a complex mixture of volatiles that changes both qualitatively and quantitatively over the different stages of bark beetle attack (Birgersson et al. 1984; Birgersson and Bergström 1989; Pettersson and Boland 2003). At early stages, we found that headspace samples from infested Norway spruce trees consisted mainly of hydrocarbon mono- and sesquiterpenes, which are released as a result of bark beetle tunneling (Phillips and Croteau 1999; Keeling and Bohlmann 2006), while in late stages of attack release of oxygenated monoterpenes increased, due to the establishment of symbiotic microorganisms such as yeasts (Birgersson et al. 1984; Leufvén et al. 1984; Leufvén and Birgersson 1987) and fungi introduced by the bark beetles (Kandasamy et al. 2016, 2021). For example, *Ophiostomatoid* fungi (*Endoconidiophora polonica*, *Grosmannia penicillata, Leptographium europhioides, Ophiostoma bicolor, O. piceae*) lining the gallery walls in bark beetle-infested trees contribute to the release of oxygenated monoterpenes such as camphor, pinocamphone, borneol, and terpinen-4-ol (Kandasamy et al. 2016, 2021). In agreement with our findings, Pettersson and Boland (2003) observed that the maximum ratio of oxygenated monoterpenes occurs in later stages of beetle attack, which coincides with the presence of late instar bark beetle larvae and appears to be an important cue for parasitoids that attack bark beetles (Pettersson 2001). In our study, we also found that infested cut trees emitted significantly higher amounts of certain volatile compounds compared to infested standing trees. According to the literature, cutting induces changes in the volatile composition, such as increased release of oxygenated monoterpenes (Strömvall and Petersson 1991; Pettersson and Boland 2003). Mechanical damage increases the release of volatile terpenes from host trees that can auto-oxidize when exposed to the air generating more oxygenated monoterpenes (Keeling and Bohlmann 2006; Benoid et al. 2021). In addition, exposed wounds can be contaminated with various types of microorganisms that can contribute to the emission of compounds from cut trees. In our study, we have not measured and compared the volatiles from non-infested cut trees and for this reason it is not possible to conclude which compounds are being produced as a direct result of the mechanical damage.

According to Hedgren et al. (2004), differences in the release rates and composition of volatiles from infested standing and infested cut trees seem to affect attraction of different *Medetera* spp. species, with the total number of *Medetera* species emerging from bark beetle-infested standing Norway spruce trees being 10 times higher than the number emerging from infested cut trees. Moreover, some *Medetera* species were present in both standing and cut Norway spruce trees, while other species only occurred in standing or in cut infested trees. In addition to the composition and release rates of compounds from infested cut or infested standing trees, the number of prey beetles, nutritional quality of the bark and visual cues such as tree orientation, bark texture, and hardness may also influence host location and oviposition by *Medetera* flies (Lawson et al. 1996; Goyer et al. 2004).

Previous studies have shown that *M. setiventris* and *M. melancholica* are attracted to components of *I. typographus* aggregation pheromone and that the attraction increases if aggregation pheromone is combined with host tree monoterpenes (α-pinene, β-pinene, and limonene) (Rudinsky et al. 1971; Hulcr et al. 2005, 2006). However, logs from infested trees have been found to be more attractive to *M. bistriata* Parent adults than a mixture of bark beetle aggregation pheromone and tree monoterpenes, indicating that additional cues, such as volatile organic compounds produced by microbial bark beetle symbionts, might play a role in host location (Williamson 1971). *Medetera signaticornis* adults arrive at freshly attacked trees almost simultaneously with bark beetles, but have also been found on attacked trees after emission of bark beetle pheromone has ceased (Lawson et al. 1997). Like *M. bistriata*, *M. signaticornis* adults may use other reliable host cues besides bark beetle aggregation pheromone and tree compounds.

Odors emitted from microorganisms living in symbiosis with bark beetles have been shown to impact the behavior of some unidentified *Medetera* species, which are more attracted to logs colonized by fungi (e.g., *Ophiostoma ips*) or a bacterial strain (*Burkholderia* sp.) than to uncolonized logs (Boone et al. 2008). Individuals of *M. signaticornis* are commonly found on Norway spruce trees infested with *I. typographus*, but have also been reported on other *Picea* and *Pinus* tree species infested with bark beetles from the genera *Dendroctunus*, *Dryocoetes*, *Scolytus*, and *Pityogenes* (Coleoptera: Curculionidae, Scolytinae) (Bickel 1985). Many of these bark beetle species, if not all, are associated with *Ophiostomatoid* fungi (Klepzig and Six 2004). Thus, volatiles from *Ophiostoma* fungi combined with tree-produced compounds may provide reliable cues for the predatory *M. signaticornis* to detect hosts throughout a bark beetle attack, even after pheromone production by the bark beetle has ceased. Microbial odors are important components of tritrophic interactions and may contribute to the attraction or repellence of predators and parasitoids to food sources or oviposition sites (Davis et al. 2013; Kandasamy et al. 2016).

Our GC-EAD studies on odors collected from freshly attacked spruce logs revealed that *M. signaticornis* males and females were able to detect several compounds produced by the host trees, bark beetles, and bark beetle associated microorganisms. The flies responded to (–)-*cis*-verbenol, ( +)-*trans*-verbenol, and myrtenol. These three compounds are produced by the bark beetle *I. typographus* (Birgersson et al. 1984; Birgersson 1989) and are detoxification products from ( ±)-α-pinene (i.e. (–)-(4*S*)-*cis*-verbenol from (–)-α-pinene, ( +)-(4*S*)-*trans*-verbenol from ( +)-α-pinene, and myrtenol from both ( +) and (–)-α-pinene), but only (–)-*cis*-verbenol is known as a pheromone component by *I. typographus* (Renwick et al. 1976; Wood 1982; Lindström et al. 1989; Blomquist et al. 2010). The flies also responded to isoterpinolene, α-pinene oxide, camphor, pinocamphone, terpinen-4-ol, myrtenal, borneol, α-terpineol, verbenone and geranyl acetone. These compounds are known to be primarily produced by microorganisms associated with *I. typographus* (Leufvén et al. 1984, 1988; Kandasamy et al. 2021). However, some compounds (e.g., terpinen-4-ol, camphor, borneol, α-terpineol and verbenone) can also be found in small amounts in the different parts (e.g., needles, bark, roots) of a healthy Norway spruce tree (Duan et al. 2020) or in other plants species (e.g., pinocamphone, camphor, borneol, α-terpineol, verbenone and geranyl acetone) (Knudsen et al. 1993). Verbenone can also be produced by many species of *Dendroctonus.* However, *Ips* beetles in general, and *I. typographus* in particular, does not produce verbenone (Francke and Vité 1983). Therefore in this case microorganisms are the most probable source of this compound. Verbenene also included in this group is not oxygenated *per se*, but may be a deoxidized product of verbenone as both compounds have a similar chemical structure and according to Blomquist et al. (2010) verbenol, verbenone and verbenene are all produced from hydroxylation of α-pinene. In addition, verbenene has not been found produced by *I. typographus* or the host tree and therefore the most probable source is microbial.

Interestingly, many of these GC-EAD active compounds are also known to be detected by other natural enemies of *I. typographus* (see Supplementary Table 4). For example, the predatory clerid beetle *Thanasimus formicarius* Linnaeus (Coleoptera: Cleridae) is attracted to bark beetle aggregation pheromone and tree monoterpenes, and possesses olfactory receptors for oxygenated monoterpenes produced by symbiotic microorganisms (e.g., camphor and pinocamphone) (Hansen 1983; Tømmerås 1985). Similarly, the Pteromalid parasitoid species *Rhopalicus tutela* Walker, *Roptrocerus mirus* Walker, and *Roptrocerus xylophagorum* Ratzeburg (Hymenoptera: Pteromalidae) respond to tree-produced compounds, but seem to be more attracted to oxygenated monoterpenes (e.g., camphor and pinocamphone) primarily produced by symbiotic fungi of the bark beetle (Pettersson 2001; Pettersson et al. 2001; Pettersson and Boland 2003). The detection of such microbial odors by different classes of natural enemies indicates that these may be crucial for location of bark beetles as prey. Therefore, further studies need to be performed to determine whether specific fungal compounds are necessary or sufficient to attract natural enemies such as *Medetera* flies.

Our field experiments with traps baited with synthetic blends of potential host cues revealed attraction for both sexes of *M. signaticornis.* Unsurprisingly, females, which use spruce trees for oviposition, were attracted in higher numbers than males. It is unclear why males are attracted to bark beetle-infested trees, but they are possibly used as meeting and mating sites by both sexes of *M. signaticornis* (Hopping 1947). Individuals of *M. ambigua* were also found in the traps, indicating that attraction was not limited to *M. signaticornis*. In the future, we will examine in more detail the attractiveness of synthetic blends for *M. signaticornis* and other *Medetera* species.

In this study, we tested and confirmed the hypothesis that *M. signaticornis* adult flies use multiple semiochemicals to detect bark beetle-infested Norway spruce trees throughout infestation. Male and female flies responded both electrophysiologically and behaviorally to several compounds emitted from host trees, bark beetles, and symbiotic microorganisms. Besides tree-produced compounds, oxygenated monoterpenes produced by symbiotic microorganisms may be a reliable cue for the predatory *M. signaticornis*, especially in the later stages of bark beetle attack when production of bark beetle pheromone has declined. Thus, the multitrophic interaction between predatory *M. signaticornis*, bark beetle, host tree, and microorganisms needs to be assessed in future studies on management and ecology of bark beetles and their natural enemies. The present study provides a sound foundation for further field work aiming to adjust the attractiveness of the synthetic blend to mimic relevant host cues. Such a blend could be used to monitor *Medetera* flies or to attract more flies to newly infested areas, increasing biological control and reducing the number of bark beetles emerging from infested trees, which in turn would reduce the economic losses to the forest sector.

### Supplementary Information

Below is the link to the electronic supplementary material.

Supplementary file1 (XLSX 11 KB)

Supplementary file2 (DOCX 30 KB)

Supplementary file3 (XLSX 122 KB)

Supplementary file4 (DOCX 33 KB)

Supplementary file5 (DOCX 32 KB)

Supplementary file6 (JPG 433 KB)

Supplementary file7 (PNG 1482 kb)

High resolution image (TIF 9715 kb)

## Data Availability

All of the data on which conclusions rely in this study are included in this published article and its supplementary information files.
