# Supplementary material for: Odors Attracting the Long-Legged Predator Medetera signaticornis Loew to Ips typographus L. Infested Norway Spruce Trees
Source: J Chem Ecol. 2023 Jan 31;49(7-8):451–64. doi: 10.1007/s10886-023-01405-6 (PMC10611644; doi:10.1007/s10886-023-01405-6)
Supplement: Supplementary file 2 — Supplementary file2 (DOCX 30 KB) [file 10886_2023_1405_MOESM2_ESM.docx]

**Supplementary Table 1:** Abundance (ng(dm^2.^s)^-1^) of compounds detected in the odour samples collected from different treatments: bark beetle non-infested trees, bark beetles infested cut and standing trees. S1-2: sites were the odour samples were collected. C1-7: order of collection (C1 corresponds to first collection, while C7 corresponds to the last/seventh collection).
