# Supplementary material for: Odors Attracting the Long-Legged Predator Medetera signaticornis Loew to Ips typographus L. Infested Norway Spruce Trees
Source: J Chem Ecol. 2023 Jan 31;49(7-8):451–64. doi: 10.1007/s10886-023-01405-6 (PMC10611644; doi:10.1007/s10886-023-01405-6)
Supplement: Supplementary file 4 — Supplementary file4 (DOCX 33 KB) [file 10886_2023_1405_MOESM4_ESM.docx]

**Supplementary Table 3** List of compounds that significantly differed between treatments, order of collection and/or sites. Treatments corresponds to infested and non-infested samples, order of collection variated from C1 to C7 (C1 corresponds to first collection while C7 corresponds to the last/seventh collection). The abundance (ng(dm^2^*s)^-1^) of compounds was compared using the multi-level pattern analysis (*Multipatt*). The *stat* indicates the strength of the association of each compound with the group tested and variates between 0 and 1. The P value provides a measure for significant difference on the abundance of the compound found between the groups tested.

|  | Treatments | | | Order of collection | | Sites |
| --- | --- | --- | --- | --- | --- | --- |
| Compounds | Significantly associated with infested trees | Significantly associated with infested cut trees | Significantly associated with infested standing trees | Significantly associated with early collection time points (C1-C2) | Significantly associated with late collection time points (C5-C7) | Significantly associated with Site 1 |
| tricyclene |  | Stat=0.17; P<0.05* |  |  |  |  |
| α-pinene | Stat=0.36; P<0.05* |  |  |  |  |  |
| α-thujene |  |  |  | Stat=0.51; P<0.01** |  |  |
| 2-methylbut-3-en-2-ol |  |  |  | Stat=0.69 P<0.001*** |  |  |
| α-fenchene | Stat=0.41; P<0.01** |  |  |  | Stat=0.58; P<0.01** |  |
| camphene | Stat=0.54; P<0.001*** | Stat= 0.41; P<0.01** |  |  | Stat=0.44; P<0.05* | Stat=0.35; P<0.01** |
| β-pinene | Stat=0.38; P<0.05* |  |  |  |  | Stat=0.35; P<0.01** |
| verbenene | Stat=0.30; P<0.05* |  |  |  |  |  |
| 2,4-thujadiene | Stat=0.41; P<0.001** | Stat=0.34; P<0.01** |  |  | Stat=0.56; P<0.01** |  |
| 3-carene |  |  |  |  |  | Stat=0.36; P<0.01** |
| β-myrcene | Stat=0.35; P<0.05* |  |  | Stat=0.55; P<0.01** |  | Stat=0.31; P<0.05* |
| α-phellandrene | Stat=0.41; P<0.01** | Stat=0.31; P<0.05* |  |  |  | Stat=0.33; P<0.01** |
| cumene | Stat=0.58; P<0.001*** | Stat=0.49; P<0.001*** |  |  | Stat=0.48; P<0.05* | Stat=0.42; P<0.001*** |
| α-terpinene | Stat=0.45; P<0.001*** | Stat=0.38; P<0.001*** |  |  |  | Stat=0.34; P<0.05* |
| limonene | Stat=0.47; P<0.01** | Stat=0.44; P<0.001*** |  |  |  | Stat=0.29; P<0.05* |
| β-phellandrene | Stat=0.36; P<0.05* |  |  | Stat=0.56; P<0.01** |  | Stat=0.32; P<0.05* |
| 2-hydroxy-1,8-cineole | Stat=0.37; P<0.01** |  |  |  |  | Stat=0.26; P<0.05* |
| ocimene | Stat=0.33; P<0.05* | Stat=0.27; P<0.05* |  |  |  | Stat=0.28; P<0.01** |
| γ-terpinene | Stat=0.40; P<0.01** | Stat=0.37; P<0.001*** |  |  |  | Stat=0.27; P<0.05* |
| p-cymene | Stat=0.57; P<0.001*** |  |  |  | Stat=0.41; P<0.05* | Stat=0.41; P<0.001*** |
| isoterpinolene | Stat=0.38; P<0.01** | Stat=0.31; P<0.05* |  |  |  | Stat=0.31; P<0.01** |
| terpinolene | Stat=0.32; P<0.05* |  |  |  |  | Stat=0.26; P<0.04* |
| sulcatone | Stat=0.32; P<0.05* | Stat=0.29; P<0.05* |  |  |  |  |
| α-pinene oxide | Stat=0.33; P=0.05* | Stat=0.33; P<0.05* |  |  |  | Stat=0.36; P<0.01** |
| nonanal | Stat=0.62; P<0.001*** | Stat=0.55; P<0.001*** |  |  |  | Stat=0.37; P<0.001*** |
| fenchone | Stat=0.52; P<0.001*** | Stat=0.43; P<0.001*** |  |  | Stat=0.56; P<0.01** | Stat=0.034; P<0.01** |
| 4-thujanol |  |  |  | Stat=0.60; P<0.01** |  |  |
| camphor | Stat= 0.62; P<0.001*** | Stat=0.54; P<0.001*** |  |  |  | Stat=0.32; P<0.01** |
| pinocamphone | Stat=0.51*; P<0.001*** | Stat=0.42; P<0.001*** |  |  |  | Stat=0.30; P<0.05* |
| pinocarvone | Stat=0.417; P<0.001*** | Stat=0.35; P<0.001*** |  |  | Stat=0.46; P<0.05* | Stat=0.35; P<0.001*** |
| bornyl acetate |  |  | Stat=0.28; P<0.05* |  |  |  |
| β-pinone | Stat=0.45; P<0.001*** | Stat=0.36; P<0.01** |  |  | Stat=0.57; P<0.01** | Stat=0.40; P<0.001*** |
| terpinen-4-ol | Stat=0.51; P<0.001*** | Stat=0.44; P<0.001*** |  |  |  | Stat=0.33; P<0.01** |
| β-caryophyllene |  |  |  | Stat=0.53; P<0.01** |  | Stat=0.27; P<0.01** |
| myrtenal | Stat=0.44; P<0.001*** | Stat=0.37; P<0.001*** |  |  | Stat=0.47; P=0.05* | Stat=0.31; P<0.001*** |
| trans-β-farnesene | Stat=0.36; P<0.01** | Stat=0.32; P<0.001*** |  |  |  | Stat=0.25; P<0.05* |
| (-)-cis-verbenol |  |  |  | Stat=0.56; P<0.01** |  |  |
| trans-pinocarveol | Stat=0.42; P<0.001*** | Stat=0.36; P<0.001*** |  |  | Stat=0.42; P<0.05* | Stat=0.32; P<0.001*** |
| 4-allylanisole |  |  |  | Stat=0.62; P<0.001*** |  |  |
| trans-verbenol |  | Stat=0.21; P<0.05* |  | Stat=0.40; P<0.05* |  |  |
| α-caryophyllene |  |  |  | Stat=0.30; P<0.01** |  | Stat=0.27; P<0.05* |
| α-terpineol | Stat=0.47; P<0.001*** | Stat=0.39; P<0.001*** |  |  |  | Stat=0.37; P<0.01** |
| borneol | Stat=0.53; P<0.001*** | Stat=0.45; P<0.001*** |  |  | Stat=0.42; P<0.05* | Stat=0.38; P<0.001*** |
| verbenone | Stat=0.47; P<0.001*** | Stat=0.38; P<0.001*** |  |  | Stat=0.41; P<0.05* | Stat=0.33; P<0.01** |
| myrtenol | Stat=0.37; P<0.001*** | Stat=0.30; P<0.05* |  |  | Stat=0.44; P<0.05* | Stat=0.31; P<0.001*** |
| cis-trans carveol | Stat=0.40; P=0.001*** | Stat=0.357; P<0.001*** |  |  |  |  |
| geranyl acetone |  |  | Stat=0.29; P<0.05* |  |  |  |
| methyl eugenol |  |  |  | Stat=0.43; P<0.05* |  |  |
