# Supplementary material for: Odors Attracting the Long-Legged Predator Medetera signaticornis Loew to Ips typographus L. Infested Norway Spruce Trees
Source: J Chem Ecol. 2023 Jan 31;49(7-8):451–64. doi: 10.1007/s10886-023-01405-6 (PMC10611644; doi:10.1007/s10886-023-01405-6)
Supplement: Supplementary file 5 — Supplementary file5 (DOCX 32 KB) [file 10886_2023_1405_MOESM5_ESM.docx]

Supplementary Table 4: Review of the electroantennographic (EAG) active compounds described for different *Ips typhographus* predator species.

|  |  | ***Thanasimus***  ***formicarius*** | ***Rhopalicus tutela*** | ***Roptrocerus xylophagorum*** | ***Roptrocerus***  ***mirus*** | ***Celoides bostrichorum*** |
| --- | --- | --- | --- | --- | --- | --- |
| **Bark beetle produced compounds** | 2-methyl-3-buten-2-ol | x |  |  |  |  |
|  | **(+)-*trans*-verbenol** | x | x |  |  |  |
|  | **(-)-*cis*-verbenol** | x |  |  |  |  |
|  | **myrtenol** |  | x | x |  | x |
|  | ipsenol | x | x |  |  |  |
|  | ipsdienol | x | x |  |  |  |
| **Compounds produced by symbiotic microorganisms** | **camphor** | **x** | **x** | **x** | **x** | **x** |
|  | **pinocamphone** | **x** | **x** |  | **x** |  |
|  | isopinocamphone |  | x | x | x | x |
|  | pinocarvone |  | x |  | x |  |
|  | trans-pinocarveol |  | x | x | x | x |
|  | **terpinen-4-ol** |  | **x** | **x** | **x** | **x** |
|  | **α-terpineol** |  | **x** | **x** | **x** | **x** |
|  | femchol |  | **x** |  |  |  |
|  | fenchone |  | **x** | **x** | **x** | **x** |
|  | **myrtenal** |  | **x** | **x** | **x** | **x** |
|  | **verbenone** | **x** | **x** | **x** | **x** | **x** |
|  | bornyl acetate |  |  | x |  |  |
|  | **borneol** |  | **x** | **x** | **x** | **x** |
|  | phenylethanol | x |  |  |  |  |
| **Host tree produced compounds** | **(±)-α-pinene** | *x* |  |  |  |  |
|  | **terpinolene** |  | x |  | x | x |
|  | p-cymene |  | x |  | x |  |
|  | β-phellandrene |  | x |  |  |  |
|  | limonene |  | x |  |  |  |
|  | linalool |  |  | x |  |  |
|  | anethol |  |  | x |  |  |
|  | 4-allylanisole |  | x |  | x | x |

*Thanasimus formicarius* (Tømmerås, 1985),  *Roptrocerus xylophagorum* (Pettersson and Boland, 2003), *Rhopalicu*s *tutela* and *Roptrocerus mirus* (Pettersson, 2001), *Coeloides bostrichorum* (Pettersson et al., 2001). Compounds in bold are EAD active on *Medetera signaticornis*.
