# Supplementary figures and images for: Odors Attracting the Long-Legged Predator Medetera signaticornis Loew to Ips typographus L. Infested Norway Spruce Trees
Source: J Chem Ecol. 2023 Jan 31;49(7-8):451–64. doi: 10.1007/s10886-023-01405-6 (PMC10611644; doi:10.1007/s10886-023-01405-6)

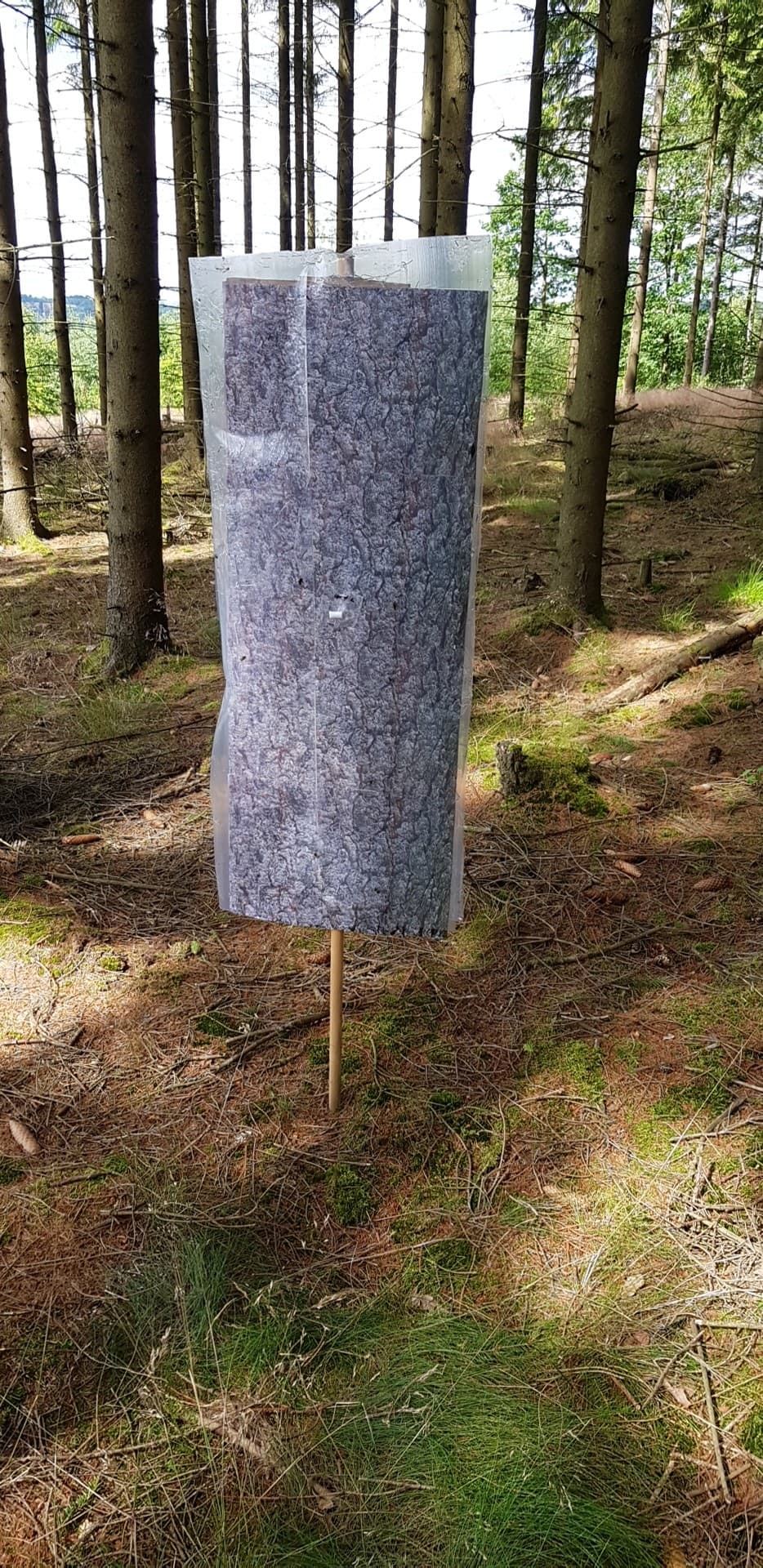

Supplement: Supplementary file 6 — Supplementary file6 (JPG 433 KB) [file 10886_2023_1405_MOESM6_ESM.jpg]

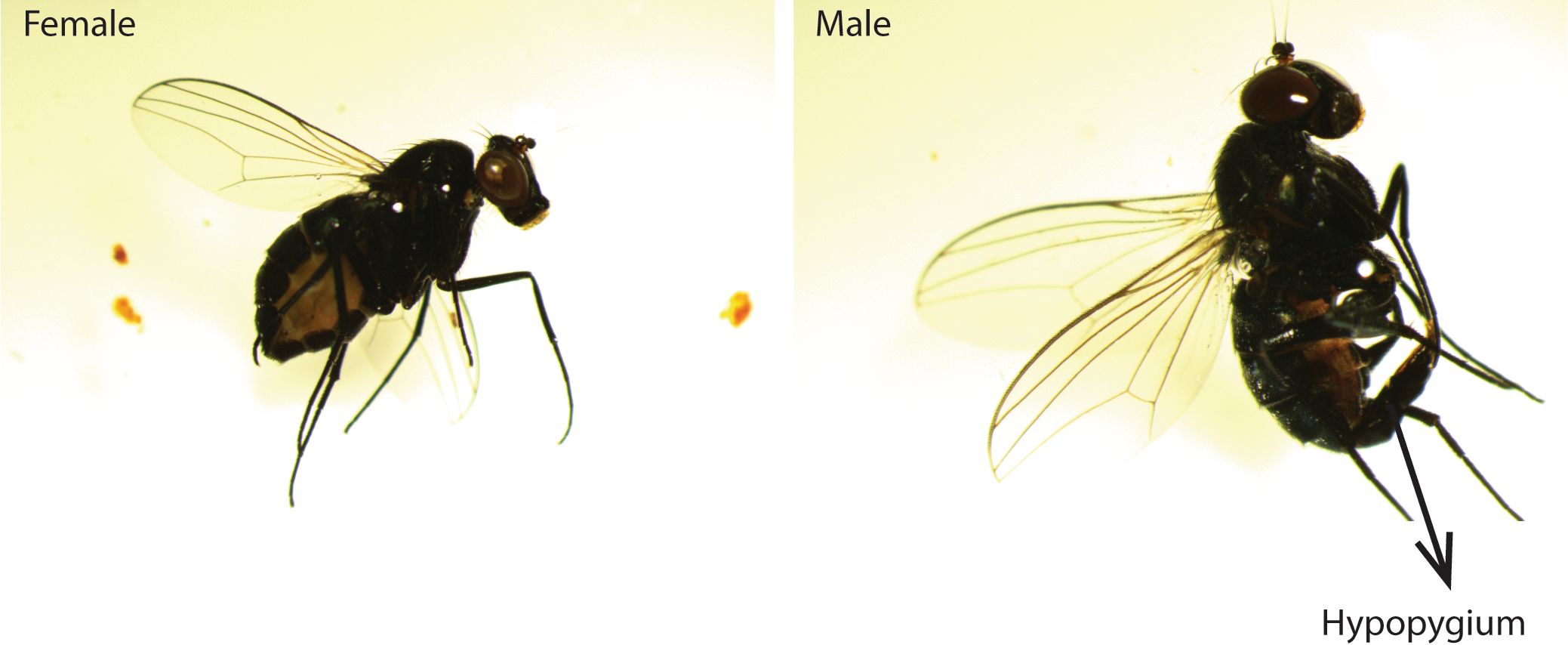

Supplement: Supplementary file 7 — Supplementary file7 (PNG 1482 kb) [file 10886_2023_1405_Fig5_ESM.png]

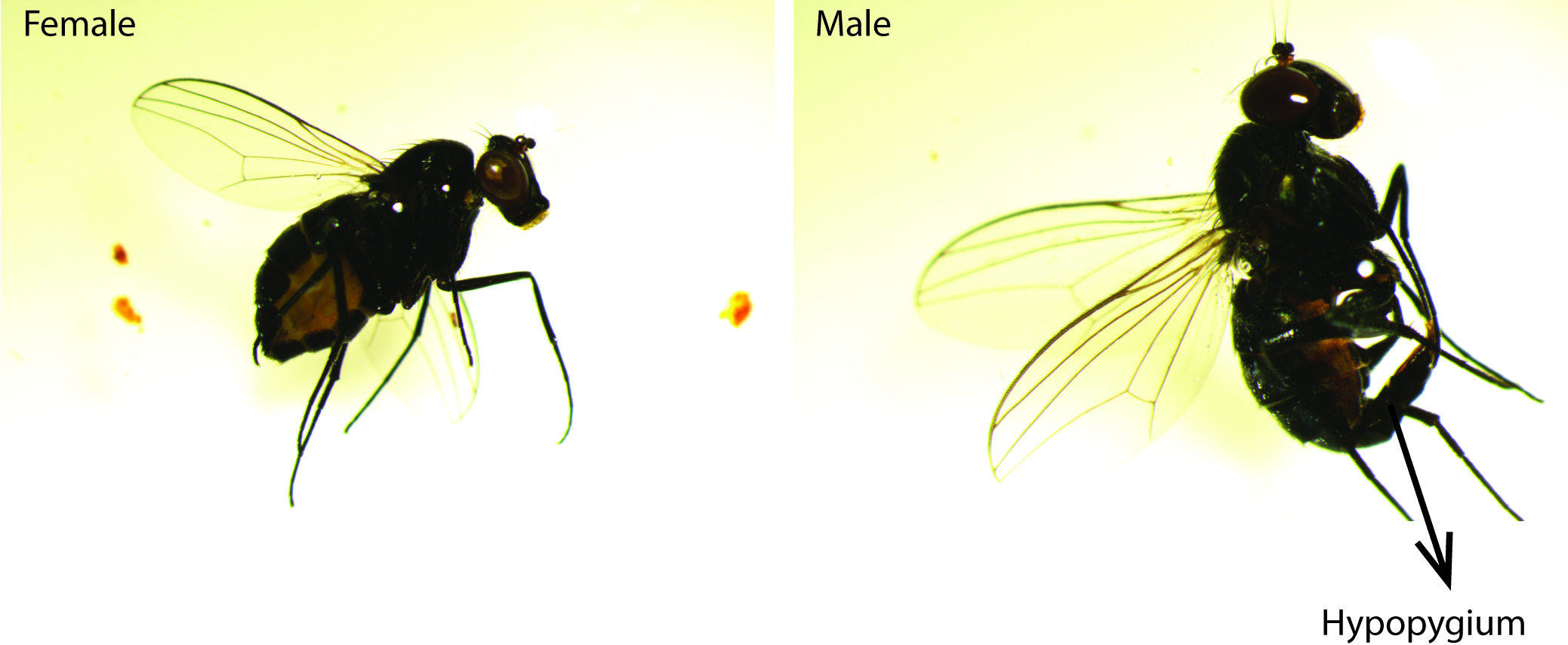

Supplement: Supplementary file 8 — High resolution image (TIF 9715 kb) [file 10886_2023_1405_MOESM7_ESM.tif]
